# Supplementary material for: Do infants and preschoolers quantify probabilities based on proportions?
Source: R Soc Open Sci. 2020 Sep 9;7(9):191751. doi: 10.1098/rsos.191751 (PMC7540750; doi:10.1098/rsos.191751)
Supplement: Supplementary material [file rsos191751supp1.pdf]

Do infants and preschoolers quantify probabilities based on proportions?

Sarah Placì[1,2,3*], Julia Fischer[1,3+], Hannes Rakoczy[2,3+]

[1]Cognitive Ethology Laboratory, German Primate Center, Kellnerweg 4, 37077 Göttingen, Germany

[2]Department of Developmental Psychology, University of Göttingen, Waldweg 26, 37073 Göttingen, Germany

[3]Leibniz ScienceCampus Primate Cognition, German Primate Center, Kellnerweg 4, 37077 Göttingen, Germany

*Corresponding Author: Sarah Placì

[+]Equal contribution

Email: sarah.placi@gmail.com

Phone +49 551 3851-0

Keywords: Probabilistic reasoning; Developmental psychology; Intuitive statistics; Child cognition

Supplementary material

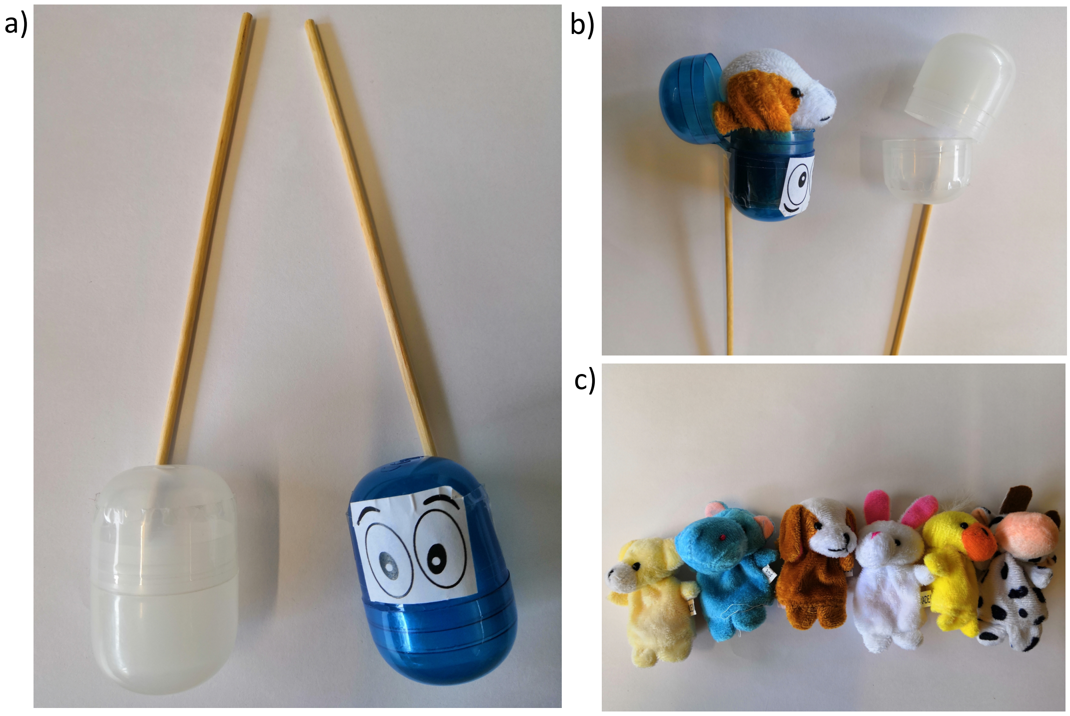

Figure S1. Objects used as rewards during the study. a) Children were presented with white and blue Kinder egg capsules. On the blue eggs were also displayed cartoon eyes. b) All blue eggs contained a fingerpuppet, all white eggs were empty. c) Different fingerpuppets used during the study.
